# Cost-effectiveness of Latent Tuberculosis Infection Screening before Immigration to Low-Incidence Countries

**DOI:** 10.3201/eid2504.171630

**Published:** 2019-04

**Authors:** Jonathon R. Campbell, James C. Johnston, Victoria J. Cook, Mohsen Sadatsafavi, R. Kevin Elwood, Fawziah Marra

**Affiliations:** University of British Columbia, Vancouver, British Columbia, Canada (J.R. Campbell, J.C. Johnston, V.J. Cook, M. Sadatsafavi, R.K. Elwood, F. Marra);; British Columbia Centre for Disease Control, Vancouver (J.C. Johnston, V.J. Cook, R.K. Elwood)

**Keywords:** tuberculosis, latent tuberculosis infection, tuberculosis and other mycobacteria, mass screening, cost–utility analysis, cost-effectiveness analysis, tuberculin test, interferon gamma release tests, migrants, immigration, Canada, bacteria

## Abstract

Prospective migrants to countries where the incidence of tuberculosis (TB) is low (low-incidence countries) receive TB screening; however, screening for latent TB infection (LTBI) before immigration is rare. We evaluated the cost-effectiveness of mandated and sponsored preimmigration LTBI screening for migrants to low-incidence countries. We used discrete event simulation to model preimmigration LTBI screening coupled with postarrival follow-up and treatment for those who test positive. Preimmigration interferon-gamma release assay screening and postarrival rifampin treatment was preferred in deterministic analysis. We calculated cost per quality-adjusted life-year gained for migrants from countries with different TB incidences. Our analysis provides evidence of the cost-effectiveness of preimmigration LTBI screening for migrants to low-incidence countries. Coupled with research on sustainability, acceptability, and program implementation, these results can inform policy decisions.

The World Health Organization (WHO) has continued working toward tuberculosis (TB) elimination, aiming to reduce the overall TB burden by ≈90% to <1 case/1 million persons in countries where TB incidence is low (low-incidence countries) (*1*). Meeting this target will require new and innovative strategies. Typically, the TB burden in low-incidence countries is highest among populations born abroad; ≈70% of TB cases occur in these populations in Canada, the United States, and much of Europe (*2*). For the most part, TB prevention in these populations has focused on identifying persons with active TB before immigration to reduce transmission after arrival. Stagnant rates of TB suggest additional methods are required to accelerate declines in TB incidence (*3*).

Universal or targeted postarrival screening for latent TB infection (LTBI) has been suggested as a method to accelerate the decline of TB (*4*); however, domestic LTBI programs exhibit suboptimal performance (*5*), are resource intensive (*6*), and may not be cost-effective (*7*). One major reason for the reduced effectiveness of postarrival LTBI screening programs is the substantial attrition in the LTBI cascade of care. More than half of patients do not reach the point of initiating treatment, which results in fewer than one fifth completing treatment (*5*).

Currently, most immigrant-receiving, low-incidence countries employ mandatory preimmigration medical exams (*8*). As part of these medical exams, a chest radiograph and medical evaluation are performed to detect TB disease before arrival or identify those who may be at increased risk for TB disease in the future; these costs are borne by the patient within their country of origin. Only a select few countries employ some form of mandated LTBI screening (*8*), and data are scarce on the yield of such programs.

A report sponsored by the US Centers for Disease Control and Prevention (Atlanta, GA, USA) suggested mandatory LTBI screening and treatment as part of routine preimmigration medical exams (*9*); however, this strategy was viewed as inequitable and unjustly coercive (*10*) and has never been employed. Alternatively, mandating and fully sponsoring only LTBI screening as a formal part of the immigration process would avoid such ethics quandaries and could substantially reduce postarrival TB incidence. Preimmigration screening coupled with postarrival follow-up could improve the yield of LTBI screening programs >2-fold (*5*), because all case-patients reporting postarrival would already have completed LTBI screening.

We evaluated the cost-effectiveness of mandating and fully sponsoring LTBI screening in prospective migrants as part of routine preimmigration medical exams, coupled with passive postarrival follow-up and treatment. We evaluated 6 strategies among migrants from 4 different TB incidence groups to determine the optimal strategy in each group for this intervention.

## Methods

### Model Overview

We chose discrete event simulation for this model because of its flexibility in varying transition times between health states in a single simulation, ability to simulate simultaneous events, and capability to model several different patient covariates. These advantages make it preferable to traditional Markov models and enable the creation of a highly representative cohort in a single simulation (*11*). We modeled new migrants, which in this evaluation refers specifically to persons who have been granted permanent resident status but have not yet become citizens of the countries they reside in. Of interest were migrants from countries belonging to 4 distinct TB incidence categories: low, <30 cases/100,000 persons/year; moderate, >30 and <100 cases/100,000 persons/year; high, >100 and <200 cases/100,000 persons/year; and very high, >200 cases/100,000 persons/year.

We further defined the 4 populations of interest by 4 covariates: patient age, bacillus Calmette-Guérin (BCG) vaccination status, chest radiograph results, and LTBI prevalence. Patient age was defined based on an age distribution of a reference cohort of permanent residents to Canada in 2014 (*12*). BCG vaccination was determined through presence of a universal BCG vaccination policy in each country of origin and adjusted by 36-year average BCG vaccine uptake (*13**–**15*). For chest radiograph, a reference cohort of permanent residents who came to Ontario during 2002–2011 was used to identify prevalence of abnormal chest radiograph results (*15*). LTBI prevalence was calibrated in each population using 2-year TB incidence in permanent resident cohorts to Ontario during 2002–2011 (*15*) and age-adjusted using the results of a meta-analysis of test-positive rates (*16*). 

We estimated LTBI prevalence using several assumptions. First, we assumed that 85% of incident TB resulted from reactivation of LTBI (*17*); second, that TB reactivation did not change over time postarrival (*18*); and last, that LTBI prevalence approximately matched reported rates of interferon-gamma release assay (IGRA) positivity in persons from each of the 4 TB incidence categories (*16*). In sum, an LTBI reactivation rate of 1.1 cases/1,000 person-years approximated literature values and yielded reasonable estimates of LTBI prevalence (*17*).

The model evaluates implementation of the intervention: preimmigration LTBI screening coupled with postarrival follow-up and treatment. The base case in this model was considered to be preimmigration TB screening without any evaluation for LTBI before or after arrival but with routine postarrival follow-up for those flagged through TB screening. We calibrated baseline TB incidence estimates and rates of postarrival follow-up to TB incidence data in permanent resident cohorts to Ontario during 2002–2011 (*15*). We considered 3 preimmigration LTBI screening options and 2 postarrival LTBI treatment options, for a total of 6 unique strategies to compare with the base case (Table 1). 

**Table 1:** Intervention strategies for screening and treatment of latent TB infection in immigrants*

Intervention strategy	Preimmigration	Postarrival if test is positive
Base case	TB screening as part of routine preimmigration medical exams, consisting of a chest radiograph, medical history, and symptom screen. If diagnosed with TB, treatment must be completed before immigrating.	Routine follow-up of those with abnormal chest radiograph results or previous TB.
TST/INH	In addition to the base case, a TST is performed at the time of the medical exam. If the result is positive (induration >10 mm) referral is made for follow-up postarrival. If the TST result is negative, no further action is taken.	Recommendation for follow-up; if patient reports for follow-up, 9-month course of INH.
TST/RIF	Same as above.	Recommendation for follow-up; at follow-up, 4-month course of RIF.
IGRA/INH	In addition to the base case, an IGRA is placed at the time of the medical exam. If the result is positive (as defined by the manufacturer) referral is made for follow-up postarrival. If the IGRA result is negative, no further action is taken. If the IGRA result is indeterminate, a second is performed; a second consecutive indeterminate is treated as a negative.	Recommendation for follow-up; if patient reports for follow-up, 9-month course of INH.
IGRA/RIF	Same as above.	Recommendation for follow-up; if patient reports for follow-up, 4-month course of RIF.
SEQ/INH	In addition to the base case, a TST is placed at the time of the medical exam. If the result is positive (as defined by an induration >10 mm) a second test is performed with an IGRA. If the subsequent IGRA result is positive (as defined by the manufacturer) referral is made for follow-up postarrival. If the initial TST is negative or if the subsequent IGRA is negative, no further action is taken. If the IGRA result is indeterminate, a second is performed; a second consecutive indeterminate is treated as a negative.	Recommendation for follow-up; at follow-up, 9-month course of INH.
SEQ/RIF	Same as above.	Recommendation for follow-up; at follow-up, 4-month course of RIF.

*No intervention required for migrants with negative results of base case screening. IGRA, interferon-gamma release assay; INH, isoniazid; RIF, rifampin; SEQ, sequential screening; TB, tuberculosis TST, tuberculin skin test.

We screened migrants with a tuberculin skin test (TST), IGRA, or sequential screening, in which persons testing positive by TST were given a confirmatory IGRA. We defined a positive TST result as an induration measuring >10 mm and a positive IGRA result using manufacturer’s recommendations, with IGRA performance being a composite measure of results from commercially available products (*19**–**21*). Although preimmigration testing was mandated, postarrival follow-up and treatment was not mandated and instead assumed to be passive, following published rates of postarrival follow-up in several countries (*22*). That is, in migrants who tested positive for LTBI, it was recommended that they attend a clinic for treatment postarrival, but no system was in place to enforce this. Those who reported for care postarrival would be treated with 9 months of isoniazid or 4 months of rifampin.

The model took a healthcare system perspective for the fully sponsored and mandated preimmigration LTBI screening: all LTBI screening costs preimmigration, along with typical postarrival costs, were the responsibility of the receiving country’s healthcare system. We used a 3% annual discount rate for costs and outcomes (*23*) and a 25-year time horizon from arrival. The main outcomes of the model were quality-adjusted life-years (QALYs), number of TB cases, and costs per 1,000 permanent residents from each of the 4 populations analyzed. These data were used to calculate the cost-effectiveness ratio, a measure that indicates the cost per additional QALY gained by an intervention strategy compared with the base case (Appendix).

**Figure 1:**
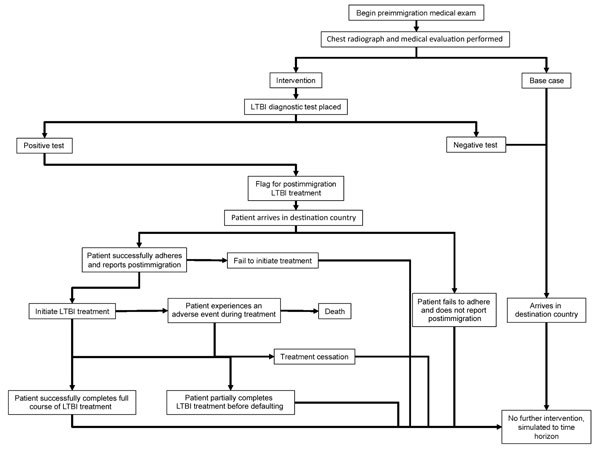
Flow structure of model used for cost-effectiveness analysis of screening and interventions of migrants for TB and LTBI. LTBI, latent tuberculosis infection; TB, tuberculosis.

A simplified model structure is displayed in Figure 1. In the intervention, migrants were given an LTBI diagnostic test along with the rest of their medical exam; those who tested positive were referred for postarrival follow-up. Those who complied with postarrival follow-up were recommended for LTBI therapy. After initiating treatment, they either completed treatment in full, partially completed treatment, or ceased due to an adverse event that may result in death. After treatment, results for all patients were simulated to the 25-year time horizon, with annual risks of TB reactivation and death. 

We made the following assumptions in the model. Those with previous TB or an abnormal chest radiograph result identified during the preimmigration medical exam were also referred for postarrival follow-up. With the intervention, all those who began screening completed it, eliminating dropout during this stage of the LTBI cascade of care. Drug-resistant TB and self-cure of LTBI were not modeled. It was assumed that all those who tested positive were offered LTBI treatment to limit extrapolation of care provider decisions. All reactivation TB cases had a 17.6% chance of causing a secondary case; further transmission was not modeled (Appendix). Modeling was completed in Simio version 8.146.14121 (Simio LLC, https://www.simio.com).

### Model Parameters

We derived model estimates from the literature or expert opinion (Table 2). A meta-analysis provided evidence for domestic LTBI program performance (*5*), therapy efficacy was derived from the literature (*24*,*27*,*28*), and adverse events were imputed from several randomized controlled trials reported in previous analysis (*24*,*25*). Diagnostic performance of LTBI screening tests was derived from systematic reviews and modeled to be the same in each country (*19**–**21*). Adherence with postarrival follow-up was estimated by reanalysis of reported data (*22*) (Appendix Figure 1). Death from tuberculosis (*3*), probability of TB therapy extension (*30*), and relapse rate (*31*) were derived from Canada sources. Life tables for Canada estimated background mortality (*32*).

**Table 2:** Model parameter estimates and values used for sensitivity analyses of intervention strategies for screening and treatment of latent TB infection in immigrants*

Parameter	**Estimate**	**Range evaluated in PSA**	**PSA distribution**	**References**
Screening parameters				
TST sensitivity	0.782	0.69–0.87	Beta(43,12)	(*19*)
TST specificity, no BCG	0.974	0.963–0.982	Beta(770,21)	(20,21)
TST specificity, BCG	0.602	0.561–0.642	Beta(239,158)	(*20**,**21*)
IGRA sensitivity	0.889	0.688–0.993	Beta(8,1)	(*19*)
IGRA specificity	0.957	0.946–0.968	Beta(900,40)	(*20**,**21*)
IGRA indeterminate†	0.06	0.05–0.07	Beta(83,1286)	(*21*)
Complete TST‡	1	Fixed	Fixed	
Complete medical evaluation§	1	Fixed	Fixed	
Population characteristics¶				
LTBI prevalence				
Very high incidence	0.3162	0.2686–0.3880	Varied with reactivation rate	(*12**,**15**–**17*)
High incidence	0.2016	0.1706–0.2464	Varied with reactivation rate	(*12**,**15**–**17*)
Moderate incidence	0.0902	0.0763–0.1102	Varied with reactivation rate	(*12**,**15**–**17*)
Low incidence	0.0159	0.0135–0.0195	Varied with reactivation rate	(*12**,**15**–**17*)
Abnormal chest radiograph results or previous TB				
Very high incidence	0.039	Fixed	Fixed	(*15*)
High incidence	0.028	Fixed	Fixed	(*15*)
Moderate incidence	0.029	Fixed	Fixed	(*15*)
Low incidence	0.008	Fixed	Fixed	(*15*)
Adherence to postarrival follow-up#	0.684	0.646–0.721	Beta(404.50,186.87)	(*22*)
Treatment parameters				
Initiate**	0.938	0.907–0.964	Beta(180.83,11.95)	(*5*)
Complete, INH	0.616	0.561–0.670	Beta(131.66,82.07)	(*5*)
Complete, RIF	0.814	0.745–0.876	Beta(76.85,17.56)	(*5*)
Adverse event, INH	0.049	0.044–0.055	Beta(249,4789)	(*24**,**25*)
Adverse event, RIF	0.021	0.018–0.025	Beta(109,4877)	(*24**,**25*)
Adverse event hospitalization	0.01	0.0005–0.03	Beta(1,99)	(*25*)
Death, INH	0.00000988	0–0.00002	Beta(2,202495)	(*26*)
LTBI risk reduction, INH	0.90	0.78–0.95	Normal(−2.3,0.5)††	(*27*)
LTBI risk reduction, RIF	0.90	0.63–0.97	Normal(−2.3,0.8)††	(*28**,**24*)
Partial risk reduction, INH	0.346	0.267–0.490	Combination of normal distributions††, ‡‡	Expert opinion, (*25*)
Partial risk reduction, RIF	0.30	0.17–0.40	Normal(−0.35,0.1)††	Expert opinion, (*24**,**28*)
Adverse event duration	7 d	0–24	Gamma(0.7,10)	Expert opinion, (*25*)
TB parameters				
Death from TB	0.0476	0.0391–0.0566	Beta(76,1523)	(*3*)
Reactivation rate	0.0011	0.0009–0.0013	Beta(90.92,82545.55)	(*15**–**17*)
Abnormal CXR risk change	3.9	3.0–4.9	Normal(1.36,0.15)††	(*29*)
Extended therapy	0.124	0.029–0.264	Beta(2.366,16.713)	Expert opinion, (*30*)
Relapse rate	0.0359	0.0197–0.0654	Normal(−3.327,0.365)††	(*30*)
Hospitalization duration	17 d	Fixed	Fixed	Expert opinion, (*30*)
Model parameters				
BCG vaccination, <30 cases	0.605	0.60–0.61	Beta(45137,29502)	(*12**,**13*)
BCG vaccination, ≥30 cases	0.998	0.997–0.999	Beta(185381,384)	(*12**,**13*)
BCG vaccination uptake	0.837	Fixed	Fixed	(*14*)
Discount rate	0.03	Fixed	Fixed	(*23*)
Time horizon	25 y	Fixed	Fixed	NA

*AE, adverse event; BCG, bacillus Calmette-Guérin; IGRA, interferon-gamma release assay; INH, isoniazid; LTBI, latent tuberculosis infection; NA, not available; PSA, probabilistic sensitivity analysis; RIF, rifampin; TST, tuberculin skin test; TB, tuberculosis. †Treated as a negative result if it occurred; was equally likely to occur in those with and without LTBI. ‡Without being mandatory, this value is 63.5% (imputed from 43.4% completing screening when 68.4% adhere with a follow-up appointment) (*5*). §Without being mandatory, this value is 78% (imputed from 43.7 of 56 individuals completing medical evaluation) (*5*). ¶Very high incidence, >200 cases/100,000; high incidence, >100 and <200 cases/100,000; moderate incidence, >30 and <100 cases/100,000; low incidence, <30 cases/100,000. #From a meta-analysis (*22*); see also Appendix (https://wwwnc.cdc.gov/EID/article/25/4/17-1630-App1.pdf). **This model assumes all who report postarrival due to a positive preimmigration LTBI diagnostic test are offered treatment. Exploratory analysis adjusts this assumption so that only the number who would complete TST screening begin treatment. ††Results from this distribution are exponentiated. ‡‡Formula: 0.33 × (Normal(−1.168,0.228)) + 0.374 × (Normal(−0.381,0.169)) + 0.293 × 1.

We derived all costs from Canada sources and assumed that the costs of screening abroad were equal to screening costs in Canada. We derived costs for LTBI treatment and screening, including drugs, screening tests, routine monitoring, and clinician time, from the British Columbia Centre for Disease Control. Adverse event costs, including hospitalization rates and time, and the cost of TB disease were as reported in the literature (*30*,*33*,*34*). We inflated all costs to 2016 Canadian dollars using consumer price indices (*35*) (Table 3).

**Table 3:** Cost and QALY estimates and values used for sensitivity analysis of intervention strategies for screening and treatment of latent TB infection in immigrants*

Parameter	**Estimate, $**	**Range evaluated in PSA**	**PSA distribution**	**References**
Costs				
Full INH treatment	992	804–1179	Triangular, 804–1179	BCCDC, (33,36)
Drug costs	181			
Nurse and clinician costs	741			
Follow-up chest radiograph	42			
Routine tests	28			
Full RIF treatment	575	464–686	Triangular, 464–686	BCCDC, (33,36)
Drug costs	98			
Nurse and clinician costs	421			
Follow-up chest radiograph	42			
Routine tests	14			
Partial INH	462	174–804	Triangular, 174–804	BCCDC, (33,36)
Partial RIF	319	178–464	Triangular, 178–464	BCCDC, (33,36)
Complete TST	31	24–38	Triangular, 24–38	BCCDC, (33,36)
TST cost	11			
Nurse costs (2 visits)	20			
Incomplete TST	21	17–25	Triangular, 17–25	BCCDC, (33,36)
IGRA	54	31–62	Triangular, 31–62	BCCDC, (33,36)
Kit and technician cost	47			
Nurse costs	7			
Chest radiograph	42	32–52	Triangular, 32–52	BCCDC, (33,36)
Cost per radiograph	35			
Nurse costs	7			
TB	20,532	7141–39,525	Gamma(4.1064,5,000)	Expert opinion, (33,34)
LTBI adverse event	732	549–916	Triangular, 549–916	(33)
Hospitalization	6,641	5305–9985	Triangular, 5,305–9,985	(30)
Death	26,933	13,079–40,788	Triangular, 13,079–40,788	(37)
QALYs				
LTBI	0.81		Assumed	(38)
Healthy	0.81	0.58–0.97	Beta(7.85,1.84)	(38)
Adverse event disutility	0.2	0.15–0.25	Triangular, ±25%	(30,33)
TB	0.69	0.08–0.24†	Beta(9,51)	(38)
Hospitalization	0.5	0.28–0.51†	Beta(19.5,30.5)	(30)
Death	0	Fixed	Fixed	Standard

*All costs are in 2016 Can $. BCCDC, British Columbia Centre for Disease Control; IGRA, interferon-gamma release assay; INH, isoniazid; LTBI, latent tuberculosis infection; PSA, probabilistic sensitivity analysis; RIF, rifampin; TB, tuberculosis; TST, tuberculin skin test. †Sampled as a percent decrement compared to healthy QALY.

We derived health utility data from a study (*38*) in Canada of migrants who reported for postarrival follow-up. We based adjustments due to adverse events or hospitalization on previous studies (*30*,*33*).

### Sensitivity Analysis

We performed a probabilistic sensitivity analysis (PSA) to capture uncertainty of model estimates using an outer sample size of 1,000 and inner sample size of 50,000 (Tables 2, 3). To guide policymakers, we created cost-effectiveness acceptability curves (CEAC) to determine the probability that the most cost-effective intervention strategy in deterministic analysis would fall below various willingness-to-pay (WTP) thresholds. Exploratory sensitivity analysis and additional probabilistic sensitivity analyses are included in the Appendix.

## Results

### Primary Results

Among migrants from moderate- to very high–incidence countries, IGRA screening coupled with postarrival rifampin treatment was the optimal intervention strategy in deterministic analysis. Sequential screening coupled with postarrival rifampin treatment was the optimal intervention strategy among migrants from low-incidence countries. Intervention strategies involving TST identified the most migrants for postarrival follow-up, whereas strategies involving sequential screening identified the fewest. Intervention strategies involving rifampin resulted in the fewest TB cases (46% reduction compared with the base case) (Table 4).

**Table 4:** Results in various TB incidence settings of implementing intervention strategies for screening and treatment of latent TB infection in immigrants*

Intervention		% Identified for post-arrival followup	Cost/1,000 persons, $	No. QALYs/1,000 persons	No. TB cases/1,000 persons	% Reduction in TB incidence	Cost per QALY gained, $**†**
Low TB incidence countries							
Base case		0.82	9,681	13,761.03	0.41	NC	NC
SEQ/RIF		4.02	60,996	13,761.30	0.26	36.87	191,889
SEQ/INH		4.02	67,309	13,761.08	0.28	32.00	1,289,335‡
IGRA/RIF		6.43	80,107	13,761.22	0.22	46.16	373,773‡
IGRA/INH		6.43	91,056	13,761.07	0.25	39.07	2,315,425‡
TST/RIF		22.99	120,910	13,760.65	0.24	40.08	Dominated
TST/INH		22.99	162,233	13,760.59	0.27	34.12	Dominated
Moderate TB incidence countries							
Base case		2.88	58,301	13,735.03	2.47	NC	NC
SEQ/RIF		11.99	121,950	13,736.36	1.57	36.52	47,561
IGRA/RIF		14.52	129,036	13,736.66	1.33	46.36	43,343
SEQ/INH		11.99	142,739	13,735.71	1.72	30.55	122,821‡
IGRA/INH		14.52	154,804	13,736.69	1.50	39.47	58,154‡
TST/RIF		38.96	206,145	13,736.84	1.46	40.77	81,548‡
TST/INH		38.96	277,998	13,735.98	1.61	34.88	230,641‡
High TB incidence countries							
Base case		2.79	122,928	13,702.56	5.39	NC	NC
SEQ/RIF		19.13	194,289	13,704.93	3.44	36.06	29,997
IGRA/RIF		23.60	199,878	13,705.48	2.91	45.99	26,350
SEQ/INH		19.13	231,835	13,704.38	3.73	30.73	59,655‡
TST/RIF		44.24	247,488	13,704.35	3.28	39.21	69,421‡
IGRA/INH		23.60	263,572	13,704.93	3.22	40.18	59,154‡
TST/INH		44.24	348,686	13,704.15	3.54	34.36	141,336‡
Very high TB incidence countries							
Base case		3.87	184,357	13,666.32	8.12	NC	NC
SEQ/RIF		27.45	263,628	13,670.25	5.18	36.23	20,165
IGRA/RIF		33.86	268,840	13,671.50	4.41	45.61	16,291
TST/RIF		49.82	318,025	13,670.32	5.62	30.76	33,403‡
SEQ/INH		27.45	318,435	13,671.23	4.86	40.16	27,296‡
IGRA/INH		33.86	337,716	13,671.02	4.97	38.82	32,657‡
TST/INH		49.82	415,877	13,669.91	5.33	34.34	64,494‡

*Very high incidence, >200 cases per 100,000; high incidence: >100 and <200 cases/100,000; moderate incidence, >30 and <100 cases/100,000; low incidence: <30 cases/100,000. IGRA, interferon-gamma release assay; INH, isoniazid; NC, not calculable; QALY, quality-adjusted life year; RIF, rifampin; SEQ, sequential screening; TB, tuberculosis; TST, tuberculin skin test. †*The cost per QALY gained is calculated in comparison to the base case. Dominated indicates that an intervention strategy has higher costs and worse outcomes compared to the base case. Costs are in CAD. **‡**This intervention strategy is strictly dominated by another intervention strategy. It is more expensive and has worse outcomes.

#### Low-Incidence Countries

For migrants from low-incidence countries, screening with TST alone resulted in a net loss in population QALYs because of poor specificity of the TST. Sequential screening, the most specific screening method, coupled with postarrival rifampin treatment yielded the lowest cost per QALY gained at $191,889. IGRA screening, the most sensitive screening method, coupled with rifampin treatment resulted in the fewest TB cases (46.2% reduction) but had a higher cost per QALY gained ($373,773) because of its lower specificity compared with that of sequential screening.

#### Moderate-Incidence Countries

For migrants from moderate-incidence countries, the optimal intervention strategy was IGRA screening coupled with postarrival rifampin treatment for those from moderate-incidence countries with a cost per QALY gained of $43,343. Sequential screening coupled with postarrival rifampin treatment was cheaper overall but had a cost per QALY gained of $47,561.

#### High-Incidence Countries

Among migrants from high-incidence countries, IGRA screening coupled with postarrival rifampin treatment was the optimal intervention strategy, at a cost per QALY gained of $26,350. Sequential screening coupled with rifampin treatment was less expensive, but also less efficient, with a cost per QALY gained of $29,997.

#### Very High–Incidence Countries

Among migrants from very high–incidence countries, IGRA screening coupled with postarrival rifampin treatment had a cost per QALY gained of $16,291 compared with the base case. Sequential screening with rifampin treatment again was slightly cheaper, resulting in a cost per QALY gained of $20,165.

### Sensitivity Analysis

**Figure 2:**
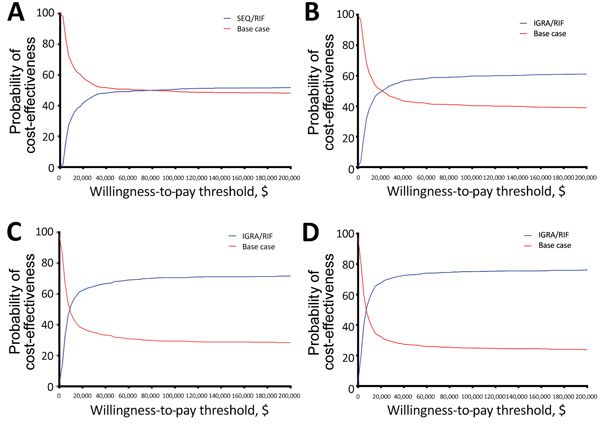
Cost-effectiveness acceptability curves of the base case of no intervention compared with intervention strategies in evaluation of screening and treatment of latent tuberculosis infection in immigrants. The graphs demonstrate the probability an option is more cost-effective at various willingness-to-pay thresholds per quality adjusted life year gained. A) Comparison of the base case with the intervention strategy of preimmigration SEQ screening coupled with postarrival RIF treatment among migrants from low-incidence countries. B) Comparison of the base case with the intervention strategy of preimmigration IGRA screening coupled with postarrival rifampin treatment among migrants from moderate-incidence countries. C) Comparison of the base case with the intervention strategy of preimmigration IGRA screening coupled with postarrival RIF treatment among migrants from high-incidence countries. D) Comparison of the base case with the intervention strategy of preimmigration IGRA screening coupled with postarrival RIF treatment among migrants from very high–incidence countries. IGRA, interferon-gamma release assay; RIF, rifampin; SEQ, sequential.

Among migrants from low-incidence countries, sequential screening coupled with postarrival rifampin treatment was the most cost-effective option in deterministic analysis. In PSA, this intervention had a probability of cost-effectiveness of 49.1% at a WTP threshold of $50,000/QALY and 50.7% at a WTP threshold of $100,000/QALY. This probability did not substantially increase past these thresholds, however, resulting in a probability of cost-effectiveness of 52% at a WTP threshold of $200,000/QALY (Figure 2, panel A).

Among migrants from moderate-, high-, and very high–incidence countries, IGRA screening coupled with postarrival rifampin treatment was the most cost-effective option in deterministic analysis. This intervention strategy at WTP thresholds of $50,000/QALY gained had probabilities of cost-effectiveness of 57.5% among migrants from moderate-incidence countries (Figure 2, panel B), 68.2% among migrants from high-incidence countries (Figure 2, panel C), and 73.2% among migrants from very high–incidence countries (Figure 2, panel D). At a WTP threshold of $100,000/QALY gained probabilities of cost-effectiveness were 59.8% among migrants from moderate-incidence countries, 70.6% among migrants from high-incidence countries, and 75.2% among migrants from very high–incidence countries.

## Discussion

The intervention of preimmigration LTBI screening followed by postarrival treatment among new migrants from countries with a TB incidence >30 cases/100,000 persons appears to be an effective method for reducing TB incidence post-arrival. The use of IGRA screening coupled with postarrival rifampin treatment provided the lowest cost-effectiveness ratio in migrants from these countries. This intervention strategy reduced TB incidence by >45% and yielded costs <$50,000/QALY gained.

Because prevalence of LTBI was low among migrants from countries with a TB incidence <30 cases/100,000 persons and specificities of LTBI diagnostic tests are imperfect, this intervention may result in a high number of uninfected persons receiving treatment unnecessarily. This finding suggests that with some strategies, the QALYs lost due to treatment side effects among those with false-positive diagnostic results may be greater than the QALYs gained by averted TB in those with true-positive diagnostic results. If screening and treatment must be performed in these low LTBI prevalence populations, more specific screening methods (i.e., sequential screening) are preferred to avoid inappropriate treatment.

Probabilistic sensitivity analysis suggests a certain degree of uncertainty in results. The behavior of CEACs as WTP thresholds increase suggests that the intervention offers small increases in population QALYs or large increases in cost in many replications. It is important to understand how well the model parameters represent the local setting when using the results of this analysis to inform evidence-based policy. These results suggest that intervention offers domestic benefits to the receiving country, but several factors need to be carefully examined. IGRA use in high-resource settings suffers from variability, in part related to several operational issues (*39*), and TST variability remains an issue (*40*). For both types of test, variability may be exacerbated in low-resource settings where LTBI prevalence rates are likely to be higher. In this model, we did not consider the costs of program initiation and maintenance; although they are outside the scope of this analysis, these costs merit careful evaluation when seeking to implement policy.

This model considered only the costs of persons who became permanent residents. The data from Canada indicated that ≈50%–60% of those who begin the process of becoming a permanent resident successfully complete it (*3*,*15*). For migrants from very high–incidence countries, assuming only half of migrants receiving preimmigration screening became permanent residents, the cost-effectiveness ratio increased 60% to ≈$26,000 when the intervention strategy was IGRA coupled with rifampin. Another consideration is the feasibility of the intervention. In a country like Canada, 2%–3% of new permanent residents are requested to follow up postarrival based on preimmigration medical exams (*3*,*15*). If the country implemented preimmigration IGRA screening for migrants from moderate- to very high–incidence countries, 17.6% would be requested to follow up postarrival (*3*,*15*). However, coupling IGRA with postarrival rifampin treatment could prevent 3.9% of all TB cases in Canada in the first year (*3*,*12*,*15*). Applied to new permanent residents to Canada in 2014, this process would increase the number requested to follow up postarrival from 6,100 to 45,800 but would result in the prevention of 61 TB cases in the first year (1 case prevented/651 additional postarrival referrals). If this process were then consistently implemented in successive cohorts in the future, it could annually prevent ≈400 TB cases.

Regardless of how preimmigration LTBI screening is implemented, investment in LTBI infrastructure in high TB incidence settings will be essential for global TB elimination. Evidence suggests that introduction of routine preimmigration TB screening in many high-income, low-incidence countries has played a role in improving infrastructure for TB programs in low-resource areas (*41*). Further introducing LTBI screening as part of these routine medical exams may have similar impact.

The cost-effectiveness of preimmigration LTBI screening and postarrival treatment has not been evaluated since 2003. Previously, Schwartzman and Menzies (*42*) examined the idea of preimmigration TST screening in addition to standard preimmigration chest radiograph coupled with post-arrival isoniazid treatment. They found the cost per TB case prevented was approximately Can $94,500. In our study, using this intervention strategy in very high incidence countries resulted in a cost per TB case prevented of approximately Can $83,000. Schwartzman et al. (*43*) later investigated the cost associated with performing a TST in all new legal immigrants from Mexico, a low-incidence country, and coupling it with postarrival isoniazid treatment. This resulted in a cost per TB case prevented of $1.2 million (2016 Can $). Using this same intervention strategy in our study resulted in a cost per TB case prevented of $1.1 million (2016 Can $). By evaluating new strategies data applied to a variety of TB incidence settings, our study represents a much-needed update to the literature.

Our analysis has several strengths. Use of discrete event simulation enabled realistic modeling of time spent in various health states, which is difficult to implement in Markov models. This type of model also allowed age-representative modeling of new migrants for application of age-adjusted LTBI prevalence. The source of most of the cost data was the British Columbia Center for Disease Control, which handles most TB cases in the province of British Columbia. This analysis estimated LTBI prevalence and abnormal chest radiograph prevalence using several years of immigration and TB data from Ontario. The data are likely to be generalizable, because Ontario accepts 40% of new permanent residents (*12*) and the data fit well with reported LTBI prevalence estimates (*16*), suggesting these parameters are reflective of long-term TB trends.

In this study, we assumed that all migrants were recommended postarrival LTBI treatment when they had a positive LTBI diagnostic test, which is not necessarily true; for some persons, the risk for serious adverse events may outweigh the benefit of treatment. Social factors and concurrent conditions may increase the risk for reactivation of LTBI. We have shown that the benefits of rifampin treatment for migrants from moderate- to very high–incidence countries who test positive by IGRA preimmigration outweigh the potential risks of adverse events. However, in practice, individual adverse-event risk is considered, and treatment may not be offered to all migrants. Further research designed to identify the specific populations who should be offered treatment would help inform future analyses.

We derived the reactivation rate of LTBI from the literature, but because many of those studies were based on TB incidence in those who were positive by TST, it is possible that the predictive value of the TST caused underestimation of true reactivation rates. Our analysis did not consider 3 months of once-weekly isoniazid and rifapentine as an LTBI treatment modality because it was not universally available. Literature data, however, suggest this modality may yield similar results to rifampin treatment (*44*). 

Our analysis used a healthcare system perspective, which does not consider costs incurred by persons experiencing the intervention (*45*). It is possible that consideration of costs and benefits from a societal perspective would change the results of this analysis; however, it is also likely that this difference would strengthen the preference for screening with IGRA, which requires only 1 visit, instead of TST, which requires 2, due to reduced absenteeism associated with IGRA testing. Costs per QALY gained may increase for all strategies if the time costs for migrants to follow up for LTBI treatment were considered. Finally, we assumed that TB reactivation was constant, which, while demonstrated previously (*18*), contradicts the common paradigm of decreasing risk over time (*46*). Where possible, we performed sensitivity analyses to view the effects our limitations may have on our results to better inform decision makers.

In conclusion, preimmigration IGRA screening coupled with postarrival rifampin treatment among migrants from countries with moderate to very high incidence of TB resulted in the lowest cost-effectiveness ratios. This evidence can be used to support policy decisions surrounding preimmigration LTBI screening in high-income, immigrant-receiving countries, when coupled with evaluations on program implementation, acceptability, and sustainability. Next steps in research should be to identify subgroups at highest risk for progression to TB disease to limit individual risk associated with LTBI treatment and improve the likelihood of feasibility and sustainability.

Appendix Additional information about cost-effectiveness of latent tuberculosis infection screening before immigration to low-incidence countries.
