# Supplementary material for: Cost-effectiveness of Latent Tuberculosis Infection Screening before Immigration to Low-Incidence Countries
Source: Emerg Infect Dis. 2019 Apr;25(4):661–71. doi: 10.3201/eid2504.171630 (PMC6433018; doi:10.3201/eid2504.171630)
Supplement: Appendix — Additional information about cost-effectiveness of latent tuberculosis infection screening before immigration to low-incidence countries. [file 17-1630-Techapp-s1.pdf]

Article DOI: https://doi.org/10.3201/eid2504.171630

Cost-effectiveness of Latent Tuberculosis Infection Screening before Immigration to Low-Incidence Countries

Appendix

Cost-effectiveness Ratio Calculation

To calculate the incremental cost-effectiveness ratio (ICER), the difference in costs between an intervention strategy ($Cost_i$) and the base case ($Cost_b$) is divided by the difference in quality-adjusted life years (QALYs) between an intervention strategy ($QALY_i$) and the base case ($QALY_b$). This is calculated through the formula:

$$ICER = \frac{Cost_i - Cost_b}{QALY_i - QALY_b}$$

This value can then be compared to a policy maker's willingness-to-pay (WTP) threshold. A value below this threshold is considered cost-effective and can be used as a component supporting new policies.

Efficiency Frontier

The efficiency frontier plots the costs and QALYs of each intervention and connects interventions that provide the next best option in terms of costs and QALYs. Interventions not on the frontier are either dominated by other interventions (i.e., they have higher costs and lower QALYs than other interventions) or are excluded due to extended dominance (i.e., a more expensive intervention provides a lower ICER). The values listed adjacent to lines along the frontier represent the ICER of going from one intervention to the next and can be used to support policy makers decisions on optimal intervention strategies.

Probability of Creating a Secondary Tuberculosis Case Calculation

We assumed that 85% of tuberculosis (TB) cases from our data source were due to reactivation of previously acquired latent TB infection (LTBI). To account for the remaining cases, we modeled the creation of secondary cases when a case of reactivation TB occurred. The formula based on this assumption is:

$$Probability\ of\ Creating\ a\ Secondary\ Case = \left(\frac{1}{0.85}\right) - 1 = 17.6\%$$

In this way, all TB cases are accounted for in this model and we minimize underestimation of overall longitudinal TB burden. Further to this, the method of modeling secondary cases will result in reductions in TB transmission as incidence of TB reactivation is reduced – in this way TB transmission was directly tied to incidence of TB reactivation.

Meta-Analysis of Reported Rates of Post-Arrival Reporting for Passive Follow-Up

A meta-analysis was performed using Stata software, version 12.1 (StataCorp, www.stata.com). To complete this, logit-transformed proportions were meta-analyzed using random-effects. The weighted pooled point estimate was back transformed into a proportion for ease of interpretation (Appendix Figure 1). The result of this meta-analysis is that 68.4% (95% CI: 64.6% to 72.1%) of those referred for passive (unenforced) post-arrival follow-up actually attend the clinic post-arrival.

Additional Sensitivity Analysis

Probabilistic Sensitivity Analysis

Probabilistic sensitivity analysis (PSA) was performed to assess the uncertainty in parameters and their impact on decisions. To complete this, probabilistic distributions are created and sampled each replication. When detailed cost data was available and known to fall within well-defined ranges, costs were modeled with a triangular distribution; when cost data was more uncertain, a gamma distribution, which can accommodate skewed cost data, was used. Beta distributions were used for most probabilities. When literature data was available and exact proportions of persons experiencing an event could be calculated, β distributions parameters

were defined as: α = number of persons experiencing event; β = number of persons not experiencing event. If this level of detail was not known, a β distribution was fit to represent reported 95% CI or means and standard deviations. In the case of treatment effect, lognormal distributions were used based on 2x2 tables of effectiveness or fit to our perceived level of uncertainty. For sampling health state utilities, we first sampled from the distribution of the healthy QALY. We assumed those with LTBI would have the same value. For TB and hospitalization QALYs, we sampled from distributions to decrement these QALYs based on the sampled healthy QALY.

The average results of the PSA are reported in Appendix Table 1. Variability can be seen through plots of the differences in costs and QALYs on a cost-effectiveness plane for the PSA of select strategies (Appendix Figure 2). The results of the PSA support the findings from the deterministic analysis: the intervention strategy of preimmigration interferon-gamma release assay (IGRA) screening coupled with postarrival follow-up and treatment with rifampin was the preferred strategy in migrants from moderate- to very high–incidence countries. Further analysis of the relationship between decisions is provided below in efficiency frontiers.

Efficiency frontiers are displayed in Appendix Figure 3. In each of the plotted frontiers, only one intervention was included on the frontier, with others excluded due to strict or extended dominance. Among migrants from low-incidence countries, only SEQ/RIF fell on the frontier, while among migrants from moderate high and very high–incidence countries, only IGRA/RIF fell on the frontier.

Exploratory Sensitivity Analysis

Various exploratory sensitivity analyses were performed. We analyzed the impact of limiting LTBI screening by age on outcomes. We then analyzed the impact certain parameters may have on cost-effectiveness including: modeling low LTBI therapy uptake postarrival, ensuring 100% adherence in postarrival follow-up, ensuring 100% adherence and participation in all steps of the LTBI cascade of care, extending the time horizon, altering TB reactivation rate, and modeling high and low estimates of costs.

Parameter changes evaluated for exploratory sensitivity analysis are listed in Appendix Table 2. In the case of varying the reactivation rate, LTBI prevalence estimates were also adjusted to reflect the expected TB incidence based on data used to calibrate these parameters in

the base analysis. This would result in nearly identical number of overall TB cases over the time horizon, but costs due to increasing or decreasing LTBI prevalence will change.

Results of exploratory analyses are presented in Appendix Tables 3–6. Only screening certain portions of new migrants based on age did not significantly impact cost per QALY gained but did lessen the overall reductions in TB incidence seen. Mandating postarrival follow-up improved the reduction in TB incidence by 40% compared with a passive system. On the contrary, if we modeled initiation of LTBI therapy at an extreme low value of 63.5%, reduction in TB incidence was reduced by ≈30%. This further impacted decisions on which intervention strategy was likely to be the most cost-effective. Fully mandating all parts of the LTBI cascade of care (i.e., all those test-positive must follow-up postarrival and complete treatment, except in cases of adverse events) increased overall costs of intervention strategies ≈40%, but overall reductions in TB incidence exceeded 80%.

Using a lifetime time horizon significantly improved cost-effectiveness of intervention strategies. Adjusting reactivation rate or costs did not significantly impact cost-effectiveness but did impact the overall cost of intervention strategies. In the case of adjusting reactivation rate, this was due to increasing or decreasing the number of persons with LTBI.

Appendix Table 1. Average PSA results of implementing intervention strategies in various TB incidence settings

Intervention	Cost/1,000, $	No. QALYs/1,000	No. TB cases/1,000	% Reduction in TB incidence	Cost/QALY gained, $†
Low TB incidence					
Base case	9,653	13,797.86	0.40	NC	NC
SEQ/RIF	60,425	13,798.51	0.27	33.76	77,885
SEQ/INH	67,006	13,798.11	0.29	28.41	228,858‡
IGRA/RIF	75,449	13,798.32	0.23	43.35	143,563‡
IGRA/INH	86,579	13,798.19	0.25	37.03	233,564‡
TST/RIF	121,260	13,798.08	0.25	38.07	498,153‡
TST/INH	163,467	13,798.06	0.27	33.63	751,114‡
Moderate TB incidence					
Base case	59,131	13,770.72	2.51	NC	NC
SEQ/RIF	121,736	13,771.95	1.62	35.45	50,590§
IGRA/RIF	125,439	13,772.73	1.38	44.92	32,938
SEQ/INH	142,037	13,772.33	1.72	31.53	51,332‡
IGRA/INH	151,623	13,772.54	1.53	39.12	50,769‡
TST/RIF	207,659	13,772.66	1.52	39.47	76,512‡
TST/INH	281,239	13,772.58	1.64	34.53	119,301‡
High TB incidence					
Base case	124,384	13,733.54	5.43	NC	NC
SEQ/RIF	195,166	13,737.25	3.52	35.16	19,047§
IGRA/RIF	198,604	13,738.15	3.04	44.01	16,093
SEQ/INH	232,435	13,736.49	3.76	30.82	36,612‡
IGRA/INH	245,804	13,737.39	3.31	38.98	31,501‡
TST/RIF	266,307	13,737.79	3.31	38.99	33,343‡
TST/INH	352,574	13,737.35	3.58	34.05	59,891‡
Very high TB incidence					
Base case	184,977	13,696.10	8.13	NC	NC
SEQ/RIF	265,405	13,701.78	5.31	34.66	14,163§

Intervention	Cost/1,000, $	No. QALYs/1,000	No. TB cases/1,000	% Reduction in TB incidence	Cost/QALY gained, $†
IGRA/RIF	268,953	13,702.93	4.58	43.67	12,299
SEQ/INH	320,020	13,700.59	5.68	30.12	30,062‡
TST/RIF	322,422	13,702.09	5.00	38.48	22,941‡
IGRA/INH	337,776	13,701.61	5.03	38.12	27,709‡
TST/INH	420,458	13,701.09	5.39	33.70	47,149‡

*All costs in 2016 CAD. Dominated: This intervention strategy has higher costs and worse outcomes compared to the base case.
CI, confidence interval; IGRA, interferon-gamma release assay; INH, isoniazid; NC, not calculable; QALY, quality-adjusted life year; RIF, rifampin; SEQ, sequential screening; TB, tuberculosis; TST, tuberculin skin test.
Very high incidence, ≥200 cases per 100,000; high incidence: ≥100 and <200 cases/100,000; moderate incidence, ≥30 and <100 cases/100,000; low incidence: <30 cases/100,000.
†The cost per QALY gained is calculated in comparison to the base case.
‡This intervention strategy is strictly dominated by another intervention strategy. It is more expensive and has worse outcomes.
§This intervention strategy is extendedly dominated by another intervention strategy. While it increases QALYs, it has a higher ICER than a more expensive intervention strategy

Appendix Table 2. Parameter changes for the exploratory sensitivity analysis*

Parameter	Original estimate	Exploratory sensitivity analysis estimate(s), range
Full INH treatment	$992	$804, $1,179
Full RIF treatment	$575	$464, $686
Partial INH treatment	$462	$174, $804
Partial RIF treatment	$319	$178, $464
Complete TST	$31	$24, $38
Incomplete TST	$21	$17, $25
IGRA	$54	$31, $62
Tuberculosis	$20,532	$7,141, $39,525
LTBI adverse event	$732	$549, $916
Hospitalization	$6641	$5,305; $9,985
Death	$26,933	$13,079; $40,788
Initiate treatment	93.8%	100%
Complete INH	61.6%	100%
Complete RIF	81.4%	100%
Annual reactivation rate†	0.11%	0.09%; 0.13%
Time horizon	25 y	Lifetime
Adherence to post-arrival follow-up	68.4%	100%

*All costs in 2016 Can $. INH, isoniazid; IGRA, interferon-gamma release assay; LTBI, latent tuberculosis infection; RIF, rifampin; TST, tuberculin skin test.
†Also changes the prevalence of LTBI.

Appendix Table 3. Results of exploratory sensitivity analyses in TB screening in migrants from low TB incidence countries*

Intervention	Cost/1,000, $	No. QALYs/1,000	No. TB cases/1,000	% Reduction in TB incidence	Cost/QALY gained, $†
Only screen persons ≤60 y of age					
Base case	9,681	13,761.03	0.41	NC	NC
SEQ/RIF	58,830	13,761.33	0.26	35.00	166,639
SEQ/INH	65,133	13,761.11	0.29	28.20	767,845‡
IGRA/RIF	77,220	13,761.25	0.23	44.02	312,764‡
IGRA/INH	87,622	13,761.10	0.26	37.10	1,243,726‡
TST/RIF	116,550	13,760.62	0.25	38.07	Dominated
TST/INH	156,102	13,760.67	0.27	33.35	Dominated
Only screen persons ≤35 y of age					
Base case	9,681	13,761.03	0.41	NC	NC
SEQ/RIF	45,484	13,761.34	0.34	16.46	118,623
SEQ/INH	49,337	13,761.11	0.35	12.80	501,351‡
IGRA/RIF	59,255	13,761.26	0.33	19.69	222,482‡
IGRA/INH	66,138	13,761.10	0.34	15.84	811,778‡
TST/RIF	89,077	13,760.62	0.35	14.71	Dominated
TST/INH	117,358	13,760.68	0.34	16.50	Dominated
Only screen persons 10–60 y of age					
Base case	9,681	13,761.03	0.41	NC	NC
SEQ/RIF	50,922	13,761.33	0.28	31.13	138,216
SEQ/INH	55,929	13,761.11	0.29	29.15	611,279‡
IGRA/RIF	66,222	13,761.25	0.24	39.86	257,727‡
IGRA/INH	75,153	13,761.10	0.27	34.48	990,386‡

Intervention	Cost/1,000, $	No. QALYs/1,000	No. TB cases/1,000	% Reduction in TB incidence	Cost/QALY gained, $†
TST/RIF	99,155	13,760.62	0.26	34.80	Dominated
TST/INH	132,435	13,760.68	0.28	31.05	Dominated
Only 63.5% initiate therapy after arrival					
Base case	9,459	13,760.96	0.41	NC	NC
SEQ/RIF	58,376	13,761.30	0.30	25.75	143,147
SEQ/INH	62,759	13,761.20	0.32	21.79	224,620‡
IGRA/RIF	75,049	13,761.22	0.28	32.26	255,693‡
IGRA/INH	82,340	13,761.16	0.29	27.98	362,842‡
TST/RIF	96,641	13,760.75	0.29	29.46	Dominated
TST/INH	124,688	13,760.68	0.31	23.79	Dominated
100% Adherence to post-arrival follow-up					
Base case	9,937	13,761.12	0.40	NC	NC
SEQ/RIF	65,283	13,761.84	0.19	52.25	77,011
SEQ/INH	74,688	13,761.48	0.23	44.11	178,931‡
IGRA/RIF	88,258	13,761.73	0.14	64.91	129,787‡
IGRA/INH	104,144	13,761.66	0.18	55.75	176,541‡
TST/RIF	158,280	13,761.05	0.17	58.17	Dominated
TST/INH	218,474	13,761.03	0.20	50.85	Dominated
No losses in the LTBI cascade of care					
Base case	10,173	13,761.32	0.39	NC	NC
SEQ/RIF	65,488	13,762.28	0.15	60.59	57,335
SEQ/INH	79,499	13,762.13	0.17	57.04	85,831‡
IGRA/RIF	88,899	13,762.27	0.08	78.12	82,958‡
IGRA/INH	112,814	13,762.16	0.10	75.41	122,324‡
TST/RIF	164,894	13,762.19	0.12	68.64	176,445‡
TST/INH	258,857	13,762.15	0.13	65.48	299,189‡
Reactivation rate is 0.9 cases/1,000 PY					
Base case	9,709	13,760.03	0.41	NC	NC
SEQ/RIF	61,978	13,760.30	0.25	38.35	195,474
SEQ/INH	68,898	13,760.08	0.28	31.52	1,324,360‡
IGRA/RIF	81,411	13,760.22	0.22	45.03	380,575‡
IGRA/INH	92,642	13,760.07	0.24	40.56	2,359,934‡
TST/RIF	121,635	13,759.59	0.24	40.74	Dominated
TST/INH	163,310	13,759.64	0.27	35.05	Dominated
Reactivation rate is 1.3 cases/1,000 PY					
Base case	9,533	13,761.67	0.40	NC	NC
SEQ/RIF	60,332	13,761.94	0.26	35.38	189,954‡
SEQ/INH	66,632	13,761.72	0.29	27.67	1,277,446‡
IGRA/RIF	79,335	13,761.86	0.22	45.67	370,447‡
IGRA/INH	89,844	13,761.71	0.25	37.93	2,285,044‡
TST/RIF	120,192	13,761.23	0.24	41.31	Dominated
TST/INH	161,282	13,761.28	0.26	34.15	Dominated
Lifetime time horizon					
Base case	13,375	20,735.49	0.57	NC	NC
SEQ/RIF	63,180	20,735.79	0.35	38.24	161,566§
SEQ/INH	69,518	20,735.76	0.39	30.40	203,916‡
IGRA/RIF	82,257	20,736.47	0.32	44.05	69,939
IGRA/INH	93,122	20,735.95	0.35	37.45	173,790‡
TST/RIF	123,069	20,734.57	0.34	40.34	Dominated
TST/INH	164,387	20,735.02	0.37	33.97	Dominated
Minimum estimated costs					
Base case	7,709	13,761.03	0.41	NC	NC
SEQ/RIF	45,709	13,761.30	0.26	36.87	142,099
SEQ/INH	51,306	13,761.08	0.28	32.00	975,411‡
IGRA/RIF	52,694	13,761.22	0.22	46.16	238,752‡
IGRA/INH	62,376	13,761.07	0.25	39.07	1,555,483‡
TST/RIF	99,230	13,760.59	0.24	40.08	Dominated
TST/INH	135,984	13,760.65	0.27	34.12	Dominated
Maximum estimated costs					
Base case	11,653	13,761.03	0.41	NC	NC
SEQ/RIF	72,938	13,761.30	0.26	36.87	229,171
SEQ/INH	79,963	13,761.08	0.28	32.00	1,528,335‡
IGRA/RIF	92,536	13,761.22	0.22	46.16	429,269‡
IGRA/INH	104,750	13,761.07	0.25	39.07	2,648,963‡
TST/RIF	142,655	13,760.59	0.24	40.08	Dominated
TST/INH	188,544	13,760.65	0.27	34.12	Dominated

Intervention	Cost/1,000, $	No. QALYs/1,000	No. TB cases/1,000	% Reduction in TB incidence	Cost/QALY gained, $†

*All costs in 2016 Can $. Dominated: This intervention strategy has higher costs and worse outcomes compared with the base case. IGRA, interferon-gamma release assay; INH, isoniazid; NC, not calculable; QALY, quality-adjusted life year; RIF, rifampin; SEQ, sequential screening; TB, tuberculosis; TST, tuberculin skin test.
†The cost per QALY gained is calculated in comparison to the base case.
‡This intervention strategy is strictly dominated by another intervention strategy. It is more expensive and has worse outcomes.
§This intervention strategy is extendedly dominated by another intervention strategy. While it increases QALYs, it has a higher ICER than a more expensive intervention strategy.

Appendix Table 4. Results of exploratory sensitivity analyses in migrants from moderate TB incidence countries*

Intervention	Cost/1,000, $	No. QALYs/1,000	No. TB cases/1,000	% Reduction in TB incidence	Cost/QALY gained, $†
Only screen persons <u><</u>60 y of age					
Base case	58,301	13,735.03	2.47	NC	NC
SEQ/RIF	118,834	13,736.66	1.64	33.66	37,076‡
IGRA/RIF	125,579	13,736.95	1.41	42.91	34,924
SEQ/INH	137,416	13,736.01	1.74	29.41	80,574§
IGRA/INH	149,533	13,736.94	1.56	36.67	47,669§
TST/RIF	199,887	13,737.13	1.54	37.55	67,186§
TST/INH	268,497	13,736.27	1.67	32.52	168,567§
Only screen persons <u><</u>35 y of age					
Base case	58,301	13,735.03	2.47	NC	NC
SEQ/RIF	98,543	13,736.30	2.09	15.25	31,697‡
IGRA/RIF	103,655	13,736.59	2.00	18.90	29,012
SEQ/INH	107,793	13,735.65	2.13	13.94	79,979§
IGRA/INH	116,023	13,736.58	2.07	16.28	37,222§
TST/RIF	159,486	13,736.77	2.04	17.56	58,010§
TST/INH	206,294	13,735.91	2.11	14.55	167,437§
Only screen persons 10–60 y of age					
Base case	58,301	13,735.03	2.47	NC	NC
SEQ/RIF	108,704	13,736.57	1.68	31.89	32,638‡
IGRA/RIF	114,669	13,736.88	1.49	39.59	30,343
SEQ/INH	125,466	13,735.94	1.79	27.63	73,548§
IGRA/INH	135,916	13,736.87	1.63	34.05	42,064§
TST/RIF	176,404	13,737.07	1.59	35.78	57,931§
TST/INH	235,861	13,736.21	1.75	29.27	150,695§
Only 63.5% initiate therapy after arrival					
Base case	58,301	13,734.92	2.50	NC	NC
SEQ/RIF	117,154	13,736.00	1.84	26.33	54,608‡
IGRA/RIF	123,323	13,736.33	1.70	32.08	46,250
SEQ/INH	130,963	13,735.79	1.93	22.58	83,767§
IGRA/INH	140,730	13,736.09	1.81	27.61	70,514§
TST/RIF	170,321	13,736.57	1.78	28.69	67,777§
TST/INH	218,771	13,736.42	1.87	25.25	107,079§
100% Adherence to post-arrival follow-up					
Base case	58,301	13,734.93	2.45	NC	NC
SEQ/RIF	130,209	13,737.12	1.18	51.75	32,935‡
IGRA/RIF	139,318	13,738.23	0.83	66.10	24,577
SEQ/INH	159,527	13,736.93	1.36	44.67	50,690§
IGRA/INH	177,134	13,737.59	1.09	55.63	44,712§
TST/RIF	262,181	13,737.91	1.02	58.50	68,474§
TST/INH	367,557	13,737.10	1.23	49.65	142,888§
No losses in the LTBI cascade of care					
Base case	60,621	13,735.17	2.33	NC	NC
SEQ/RIF	127,849	13,737.68	0.92	60.38	26,863‡
IGRA/RIF	136,690	13,738.68	0.52	77.51	21,717
SEQ/INH	169,175	13,737.77	0.98	57.87	41,819§
IGRA/INH	188,938	13,738.41	0.60	74.47	39,639§
TST/RIF	269,509	13,738.19	0.76	67.35	69,219§
TST/INH	429,117	13,737.59	0.81	65.19	152,307§
Reactivation rate is 0.9 cases/1,000 PY					
Base case	59,005	13,729.18	2.50	NC	NC
SEQ/RIF	127,864	13,730.85	1.57	37.06	41,217‡
IGRA/RIF	135,775	13,731.33	1.34	46.46	35,627
SEQ/INH	150,721	13,730.75	1.71	31.77	58,282§
IGRA/INH	164,567	13,731.44	1.50	40.17	46,683§

Intervention	Cost/1,000, $	No. QALYs/1,000	No. TB cases/1,000	% Reduction in TB incidence	Cost/QALY gained, $†
TST/RIF	209,711	13,731.83	1.47	41.11	56,717§
TST/INH	283,226	13,731.15	1.62	35.31	113,602§
Reactivation rate is 1.3 cases/1,000 PY					
Base case	57,807	13,738.64	2.45	NC	NC
SEQ/RIF	117,950	13,741.03	1.56	36.30	25,192‡
IGRA/RIF	124,326	13,741.33	1.33	45.98	24,716
SEQ/INH	136,278	13,740.49	1.68	31.34	42,500§
IGRA/INH	147,736	13,740.48	1.49	39.45	48,846§
TST/RIF	203,596	13,740.95	1.46	40.40	63,063§
TST/INH	274,258	13,740.54	1.60	34.62	114,013§
Lifetime time horizon					
Base case	80,521	20,589.15	3.42	NC	NC
SEQ/RIF	135,403	20,591.19	2.21	35.31	26,834‡
IGRA/RIF	141,253	20,592.00	2.42	29.17	21,301
SEQ/INH	155,841	20,591.14	1.89	44.81	37,751§
IGRA/INH	166,455	20,591.74	2.11	38.38	33,183§
TST/RIF	219,818	20,591.06	2.07	39.51	72,744§
TST/INH	291,198	20,590.12	2.26	33.75	215,855§
Minimum estimated costs					
Base case	46,426	13,735.03	2.47	NC	NC
IGRA/RIF	92,622	13,736.66	1.33	46.36	28,307
SEQ/RIF	93,356	13,736.36	1.57	36.52	35,068§
SEQ/INH	111,521	13,735.71	1.72	30.55	94,686§
IGRA/INH	115,204	13,736.65	1.50	39.47	42,470§
TST/RIF	169,528	13,736.84	1.46	40.77	67,901§
TST/INH	233,240	13,735.98	1.61	34.88	196,119§
Maximum estimated costs					
Base case	70,178	13,735.03	2.47	NC	NC
SEQ/RIF	144,900	13,736.36	1.57	36.52	55,836‡
IGRA/RIF	150,482	13,736.66	1.33	46.36	49,206
SEQ/INH	168,312	13,735.71	1.72	30.55	142,743
IGRA/INH	179,434	13,736.65	1.50	39.47	67,465§
TST/RIF	242,876	13,736.84	1.46	40.77	95,258§
TST/INH	322,868	13,735.98	1.61	34.88	265,276§

*All costs in 2016 Can $. IGRA, interferon-gamma release assay; INH, isoniazid; NC, not calculable; QALY, quality-adjusted life year; RIF, rifampin; SEQ, sequential screening; TB, tuberculosis; TST, tuberculin skin test.
†The cost per QALY gained is calculated in comparison to the base case.
‡ This intervention strategy is extendedly dominated by another intervention strategy. While it increases QALYs, it has a higher ICER than a more expensive intervention strategy.
§This intervention strategy is strictly dominated by another intervention strategy. It is more expensive and has worse outcomes.

Appendix Table 5. Results of exploratory sensitivity analyses in migrants from high TB incidence countries*

Intervention	Cost/1,000, $	No. QALYs/1,000	No. TB cases/1,000	% Reduction in TB incidence	Cost/QALY gained, ($)†
Only screen persons ≤60 y of age					
Base case	122,928	13,702.56	5.39	NC	NC
SEQ/RIF	189,766	13,703.94	3.57	33.80	48,452‡
IGRA/RIF	195,130	13,705.08	3.08	42.80	28,627
SEQ/INH	223,538	13,703.44	3.80	29.51	113,251§
IGRA/INH	239,543	13,704.43	3.43	36.35	62,167§
TST/RIF	256,075	13,705.02	3.34	38.07	54,092*
TST/INH	336,983	13,704.39	3.66	32.05	117,017§
Only screen persons ≤35 y of age					
Base case	122,928	13,702.56	5.39	NC	NC
SEQ/RIF	162,376	13,703.32	4.56	15.42	51,575‡
IGRA/RIF	166,890	13,703.57	4.36	19.13	43,327
SEQ/INH	177,765	13,703.19	4.64	13.78	86,006§
IGRA/INH	186,811	13,703.28	4.49	16.73	88,102§
TST/RIF	213,873	13,703.41	4.44	17.59	107,073§
TST/INH	263,567	13,703.20	4.60	14.65	219,339§
Only screen persons 10–60 y of age					
Base case	122,928	13,702.56	5.39	NC	NC
SEQ/RIF	179,377	13,704.28	3.70	31.33	32,716‡
IGRA/RIF	183,730	13,705.06	3.24	39.78	24,321
SEQ/INH	210,766	13,703.81	3.94	26.87	70,087§

Intervention	Cost/1,000, $	No. QALYs/1,000	No. TB cases/1,000	% Reduction in TB incidence	Cost/QALY gained, ($)†
IGRA/INH	223,488	13,704.55	3.56	33.89	50,319§
TST/RIF	235,007	13,704.25	3.50	34.96	66,195§
TST/INH	304,261	13,704.11	3.77	30.10	116,849§
Only 63.5% initiate therapy after arrival					
Base case	123,493	13,701.90	5.46	NC	NC
SEQ/RIF	187,956	13,703.54	4.02	26.37	39,248
IGRA/RIF	192,897	13,703.54	3.70	32.24	42,170
SEQ/INH	213,393	13,703.21	4.22	22.66	68,782§
IGRA/INH	224,618	13,703.45	3.93	28.10	65,044§
TST/RIF	230,606	13,703.38	3.91	28.37	72,197§
TST/INH	287,689	13,703.11	4.12	24.65	135,974§
100% adherence to post-arrival follow-up					
Base case	123,590	13,701.71	5.32	NC	NC
SEQ/RIF	205,198	13,705.26	2.57	51.59	22,981‡
IGRA/RIF	213,881	13,706.71	1.82	65.71	18,066
SEQ/INH	260,097	13,704.83	3.01	43.49	43,784§
IGRA/INH	283,398	13,705.64	2.35	55.82	40,719§
TST/RIF	316,894	13,706.31	2.24	57.87	42,089§
TST/INH	441,135	13,705.29	2.71	49.10	88,850§
No losses in the LTBI cascade of care					
Base case	123,337	13,702.66	5.13	NC	NC
SEQ/RIF	199,723	13,706.07	2.05	59.95	22,403‡
IGRA/RIF	206,838	13,707.94	1.16	77.37	15,795
SEQ/INH	274,295	13,705.94	2.19	57.32	45,942§
IGRA/INH	301,381	13,706.97	1.32	74.20	41,269§
TST/RIF	318,782	13,706.36	1.66	67.68	52,776§
TST/INH	501,470	13,705.89	1.80	64.93	116,949§
Reactivation rate is 0.9 cases/1,000 PY					
Base case	123,878	13,689.10	5.42	NC	NC
SEQ/RIF	207,013	13,692.31	3.44	36.44	25,889‡
IGRA/RIF	215,001	13,692.95	2.93	45.93	23,645
SEQ/INH	249,627	13,691.78	3.72	31.35	46,868§
IGRA/INH	269,077	13,691.92	3.26	39.73	51,459§
TST/RIF	271,148	13,692.32	3.21	40.72	45,759§
TST/INH	359,562	13,691.89	3.51	35.11	84,512§
Reactivation rate is 1.3 cases/1,000 PY					
Base case	122,759	13,710.67	5.38	NC	NC
SEQ/RIF	185,440	13,713.34	3.43	36.21	23,481‡
IGRA/RIF	190,194	13,713.62	2.92	45.75	22,844
SEQ/INH	218,459	13,712.71	3.69	31.42	47,077§
IGRA/INH	232,647	13,713.21	3.28	39.10	43,315§
TST/RIF	257,754	13,713.51	3.20	40.65	47,589§
TST/INH	340,390	13,713.27	3.52	34.57	83,627§
Lifetime time horizon					
Base case	170,849	20,402.30	7.49	NC	NC
SEQ/RIF	224,283	20,407.15	4.82	35.70	11,020‡
IGRA/RIF	225,833	20,408.27	4.06	45.77	9,210
SEQ/INH	261,944	20,405.53	5.24	30.06	28,166§
TST/RIF	275,189	20,405.94	4.61	38.44	28,678§
IGRA/INH	291,260	20,406.55	4.51	39.84	28,311§
TST/INH	377,756	20,404.24	4.97	33.69	106,493§
Minimum estimated costs					
Base case	97,691	13,702.56	5.39	NC	NC
IGRA/RIF	150,526	13,705.48	2.91	45.99	18,093
SEQ/RIF	151,483	13,704.93	3.44	36.06	22,612§
SEQ/INH	184,273	13,704.38	3.73	30.73	47,426§
IGRA/INH	192,125	13,704.35	3.28	39.21	52,631§
TST/RIF	215,987	13,704.93	3.22	40.18	49,754§
TST/INH	291,076	13,704.15	3.54	34.36	121,070§
Maximum estimated costs					
Base case	148,167	13,702.56	5.39	NC	NC
SEQ/RIF	230,715	13,704.93	3.44	36.06	34,699‡
IGRA/RIF	234,294	13,705.48	2.91	45.99	29,493
SEQ/INH	273,020	13,704.38	3.73	30.73	68,389§
IGRA/INH	287,910	13,704.35	3.28	39.21	77,883§
TST/RIF	311,276	13,704.93	3.22	40.18	68,602§
TST/INH	406,415	13,704.15	3.54	34.36	161,677§

Intervention	Cost/1,000, $	No. QALYs/1,000	No. TB cases/1,000	% Reduction in TB incidence	Cost/QALY gained, ($)†

*All costs in 2016 Can $. IGRA, interferon-gamma release assay; INH, isoniazid; NC, not calculable; QALY, quality-adjusted life year; RIF, rifampin; SEQ, sequential screening; TB, tuberculosis; TST, tuberculin skin test.
†The cost per QALY gained is calculated in comparison to the base case.
‡This intervention strategy is extendedly dominated by another intervention strategy. While it increases QALYs, it has a higher ICER than a more expensive intervention strategy.
§This intervention strategy is strictly dominated by another intervention strategy. It is more expensive and has worse outcomes.

Appendix Table 6. Results of exploratory analyses in migrants from very high TB incidence countries*

Intervention	Cost/1,000, $	No. QALYs/1,000	No. TB cases/1,000	% Reduction in TB incidence	Cost/QALY gained, $†
Only screen persons ≤60 y of age					
Base case	184,357	13,666.32	8.12	NC	NC
SEQ/RIF	257,138	13,670.07	5.36	33.99	19,422‡
IGRA/RIF	262,010	13,671.02	4.64	42.77	16,507
SEQ/INH	307,723	13,669.52	5.78	28.79	38,514§
TST/RIF	310,389	13,670.59	5.05	37.82	29,494§
IGRA/INH	325,585	13,669.95	5.16	36.45	38,909§
TST/INH	402,151	13,669.61	5.52	32.05	66,187§
Only screen persons ≤35 y of age					
Base case	184,357	13,666.32	8.12	NC	NC
SEQ/RIF	223,125	13,668.52	6.89	15.08	17,593‡
IGRA/RIF	226,103	13,668.80	6.55	19.29	16,848
SEQ/INH	244,909	13,667.52	7.03	13.44	50,260§
IGRA/INH	255,309	13,668.30	6.80	16.28	35,749§
TST/RIF	265,651	13,668.53	6.74	16.94	36,847§
TST/INH	316,918	13,667.99	6.92	14.77	79,512§
Only screen persons 10–60 y of age					
Base case	184,357	13,666.32	8.12	NC	NC
SEQ/RIF	246,752	13,670.05	5.57	31.33	16,711‡
IGRA/RIF	250,991	13,671.01	4.93	39.31	14,206
TST/RIF	290,406	13,670.58	5.28	34.94	24,898§
SEQ/INH	292,011	13,669.51	5.94	26.85	33,753§
IGRA/INH	307,714	13,669.93	5.35	34.05	34,114§
TST/INH	370,423	13,669.60	5.65	30.38	56,781§
Only 63.5% initiate therapy after arrival					
Base case	183,165	13,667.06	8.14	NC	NC
SEQ/RIF	255,496	13,669.71	6.08	25.22	27,222‡
IGRA/RIF	260,008	13,670.45	5.61	31.07	22,661
TST/RIF	286,279	13,669.35	5.85	28.10	44,932§
SEQ/INH	291,905	13,669.14	6.36	21.85	52,130§
IGRA/INH	305,683	13,669.01	5.94	26.93	62,670§
TST/INH	352,634	13,669.30	6.19	23.92	75,655§
100% Adherence to post-arrival follow-up					
Base case	185,064	13,667.21	8.01	NC	NC
SEQ/RIF	277,526	13,672.40	3.86	51.83	17,814‡
IGRA/RIF	286,742	13,673.59	2.75	65.72	15,931
SEQ/INH	356,943	13,671.03	4.50	43.88	45,057§
TST/RIF	369,167	13,673.42	3.36	58.09	29,667§
IGRA/INH	386,849	13,672.66	3.53	56.00	37,031§
TST/INH	512,140	13,672.17	4.08	49.11	65,978§
No losses in the LTBI cascade of care					
Base case	184,268	13,668.16	7.73	NC	NC
SEQ/RIF	269,103	13,674.32	3.10	59.97	13,783‡
IGRA/RIF	275,614	13,675.78	1.75	77.33	11,997
TST/RIF	366,839	13,675.63	2.52	67.46	24,447§
SEQ/INH	376,306	13,674.18	3.27	57.66	31,918§
IGRA/INH	411,237	13,675.57	1.97	74.47	30,634§
TST/INH	571,667	13,674.53	2.71	65.00	60,899§
Reactivation rate is 0.9 cases/1,000 PY					
Base case	185,203	13,646.81	8.14	NC	NC
SEQ/RIF	283,301	13,651.14	5.19	36.26	22,626‡
IGRA/RIF	291,983	13,651.90	4.43	45.51	20,962
TST/RIF	330,139	13,651.84	4.85	40.39	28,775§
SEQ/INH	346,376	13,650.64	5.63	30.82	42,038§
IGRA/INH	370,889	13,651.29	4.96	39.11	41,386§
TST/INH	433,739	13,651.04	5.34	34.41	58,669§
Reactivation rate is 1.3 cases/1,000 PY					
Base case	181,988	13,680.54	8.02	NC	NC

Intervention	Cost/1,000, $	No. QALYs/1,000	No. TB cases/1,000	% Reduction in TB incidence	Cost/QALY gained, $†
SEQ/RIF	250,392	13,684.51	5.19	35.28	17,215‡
IGRA/RIF	252,612	13,684.78	4.39	45.28	16,672
SEQ/INH	297,483	13,682.75	5.57	30.53	52,289§
TST/RIF	309,989	13,684.00	4.84	39.65	36,943§
IGRA/INH	313,436	13,683.69	4.91	38.75	41,738§
TST/INH	403,690	13,683.21	5.32	33.63	83,081§
Lifetime time horizon					
Base case	257,337	20,201.68	11.34	NC	NC
SEQ/RIF	309,830	20,208.76	7.29	35.77	7,415‡
IGRA/RIF	309,977	20,212.11	6.23	45.09	5,046
TST/RIF	362,091	20,207.77	7.90	30.38	17,210§
SEQ/INH	364,532	20,208.76	6.84	39.70	15,141§
IGRA/INH	378,284	20,208.92	7.00	38.33	16,718§
TST/INH	459,696	20,207.51	7.48	34.03	34,692§
Minimum estimated costs					
Base case	146,557	13,666.32	8.12	NC	NC
IGRA/RIF	206,883	13,671.50	4.41	45.61	11,632
SEQ/RIF	207,111	13,670.25	5.18	36.23	15,404§
SEQ/INH	254,658	13,670.32	5.62	30.76	27,013§
TST/RIF	260,362	13,671.23	4.86	40.16	23,169§
IGRA/INH	267,040	13,671.02	4.97	38.82	25,656§
TST/INH	346,126	13,669.91	5.33	34.34	55,593§
Maximum estimated costs					
Base case	222,159	13,666.32	8.12	NC	NC
SEQ/RIF	313,033	13,670.25	5.18	36.23	23,117‡
IGRA/RIF	315,886	13,671.50	4.41	45.61	18,073
SEQ/INH	374,284	13,670.32	5.62	30.76	38,015§
TST/RIF	376,642	13,671.23	4.86	40.16	31,450§
IGRA/INH	393,477	13,671.02	4.97	38.82	36,481§
TST/INH	485,765	13,669.91	5.33	34.34	73,432§

*All costs in 2016 Can $. IGRA, interferon-gamma release assay; INH, isoniazid; NC, not calculable; QALY, quality-adjusted life year; RIF, rifampin; SEQ, sequential screening; TB, tuberculosis; TST, tuberculin skin test.
†The cost per QALY gained is calculated in comparison to the base case.
‡This intervention strategy is extendedly dominated by another intervention strategy. While it increases QALYs, it has a higher ICER than a more expensive intervention strategy.
§This intervention strategy is strictly dominated by another intervention strategy. It is more expensive and has worse outcomes.

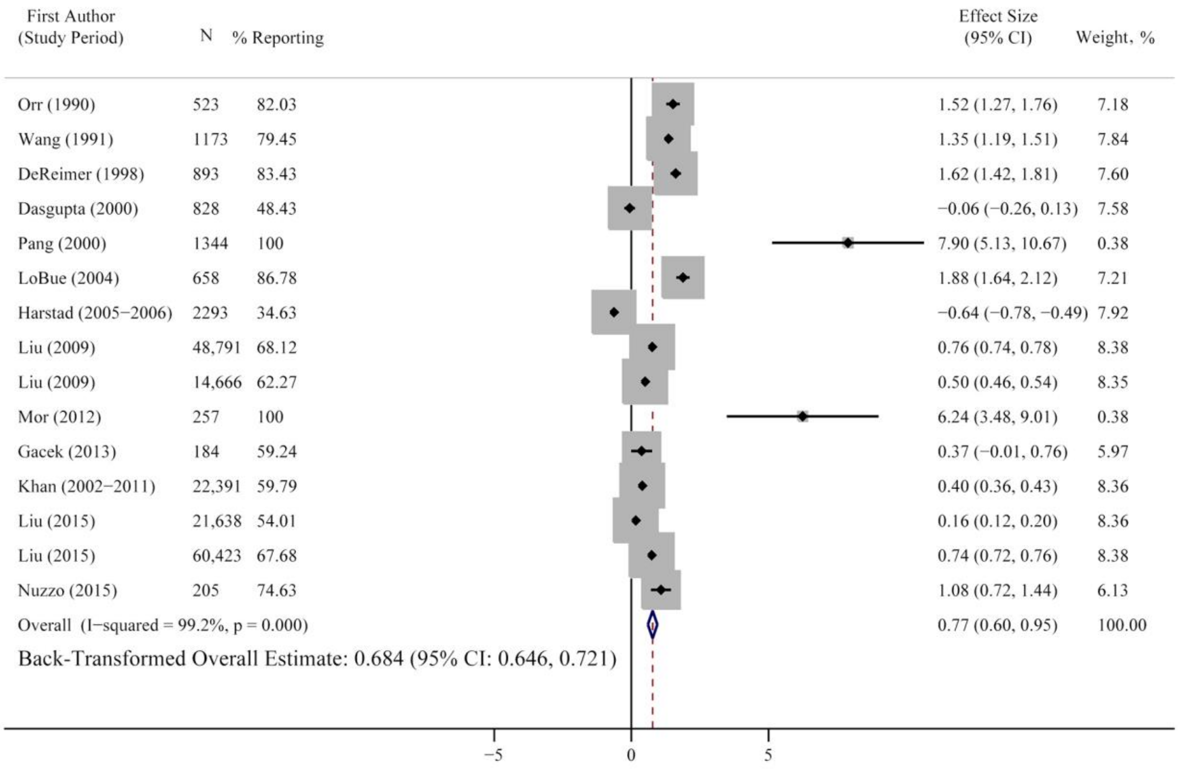

Appendix Figure 1. Meta-analysis of adherence with a request for post-arrival follow-up. This was used to inform the proportion who report for LTBI treatment post-arrival.

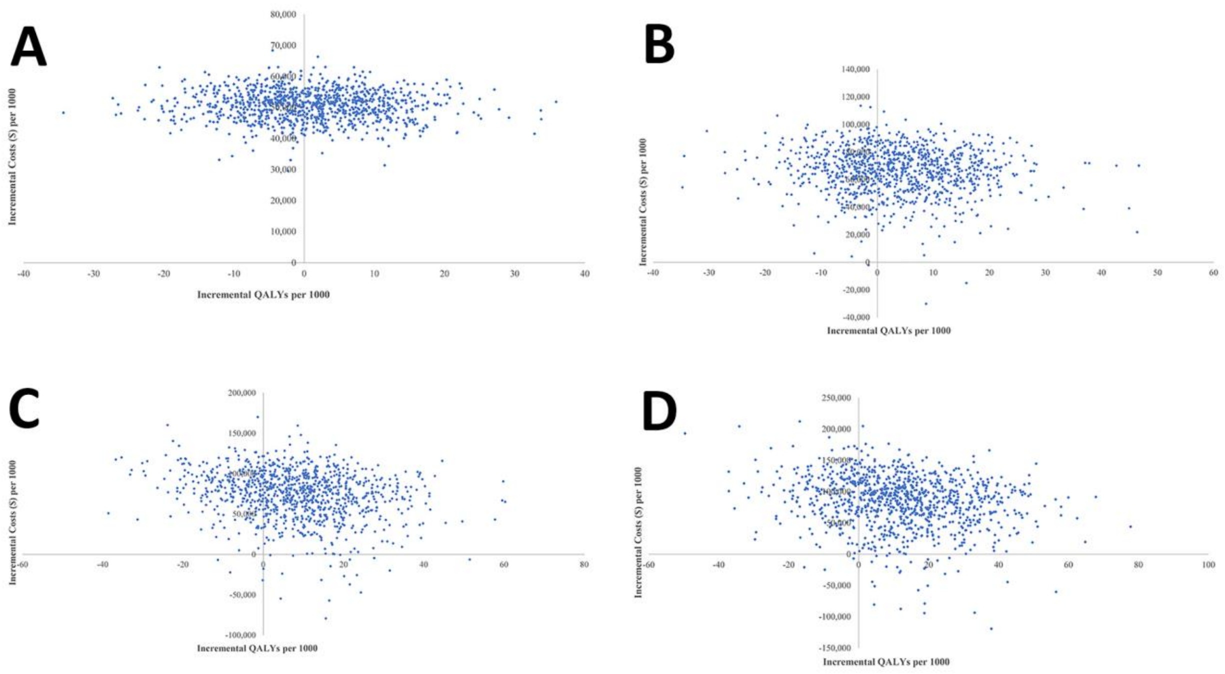

Appendix Figure 2. Cost-effectiveness planes demonstrating the variability in probabilistic sensitivity analysis replications for select intervention strategies in migrants from low incidence (A), moderate incidence (B), high incidence (C), and very high TB incidence (D) countries.

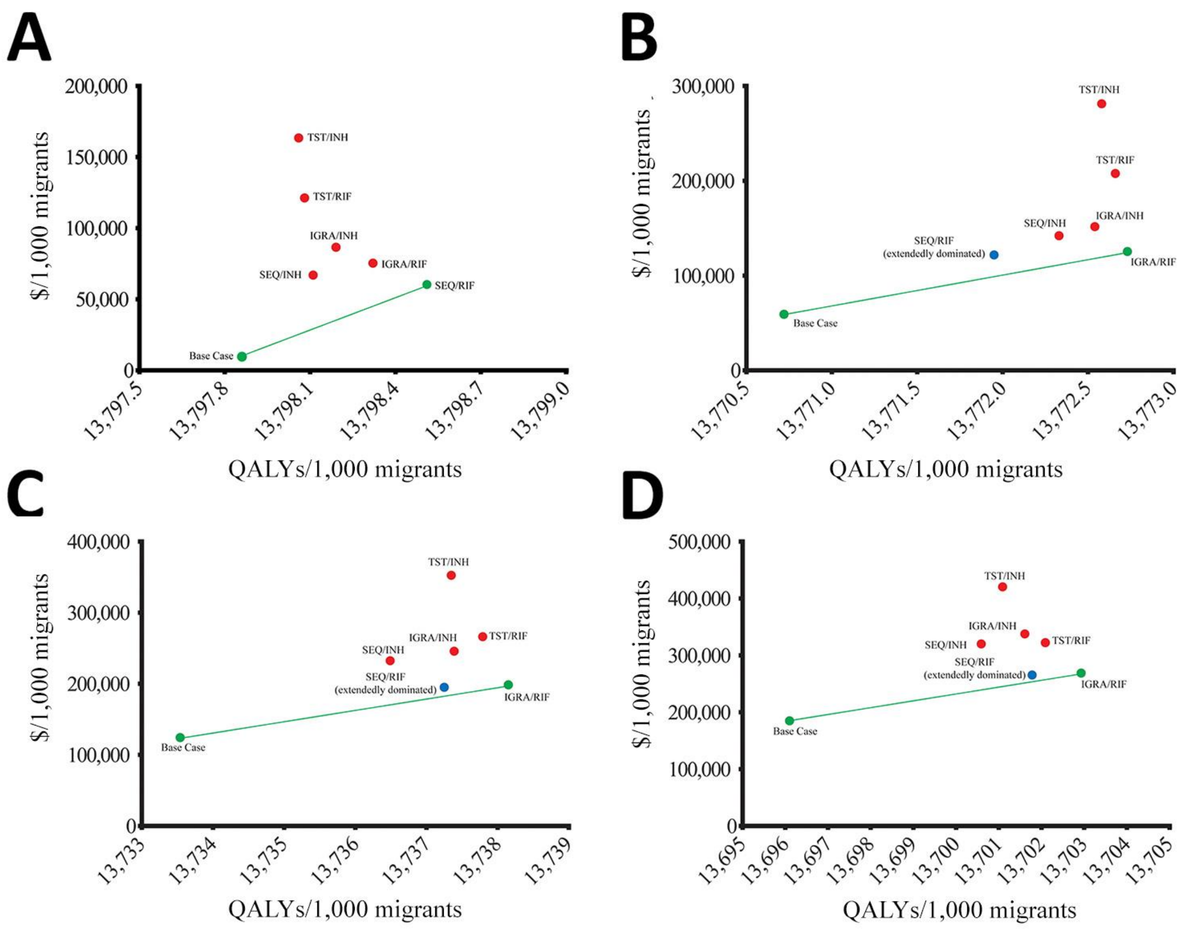

Appendix Figure 3. Efficiency frontier of population QALYs versus population costs among migrants from low (A), moderate (B), high (C), and very high (D) TB incidence countries. The frontier is read from left to right, with intervention strategies connected if they fall on the frontier. Those subsequent to the initial intervention strategy have an increased cost, but an increased benefit, and represent the next best value at increasing funding thresholds. The slope between 2 connected intervention strategies represents cost-effectiveness: a steeper slope represents poorer cost-effectiveness, while a shallow slope represents better cost-effectiveness. An intervention strategy that is extendedly dominated has a higher cost-per QALY gained and fewer population QALYs than the subsequent intervention on the frontier and is therefore less efficient. IGRA, interferon-gamma release assay; INH, isoniazid; QALY, quality-adjusted life year; RIF, rifampin; SEQ, sequential screening; TST, tuberculin skin test.
